# Dental iron precipitates in patients with Type 2 diabetes

**DOI:** 10.1002/cre2.150

**Published:** 2018-12-05

**Authors:** Miguel Angel Ortiz‐Arrambide, Karla Isabel Juarez‐Ibarra, Guadalupe Ismael Malagón‐Santiago, Norma Cruz‐Fierro, Myriam Angelica De La Garza‐Ramos

**Affiliations:** ^1^ Facultad de Odontologia Universidad Autonoma de Nuevo Leon Monterrey Mexico; ^2^ Centro de Investigación y Desarrollo en Ciencias de la Salud (CIDICS) Universidad Autonoma de Nuevo Leon Monterrey Mexico

**Keywords:** dentin, diabetes mellitus, iron overload, precipitation

## Abstract

Diabetes mellitus (DM) is a frequent worldwide disease. There are currently more than 46 million people who suffer this disease in North America and the Caribbean. The objective of this study was to determine if there is an association between DM and the presence of iron precipitates (Fe^2+^) in dental structure. The third molar was extracted for reasons that merit extraction from 40 individuals with and without DM to analyze dentin tissue. Horizontal and longitudinal slices of tooth samples were made and later stained with 10% potassium cyanoferrate. The samples were observed by optical microscope to identify basophilic elements. A nonparametric Spearman correlation was performed to find an association between the quantitative (gender, group, and dentinal tissue) and qualitative variables (gender). The Mann–Whitney U test was used to find differences in the means of the nonparametric variables in two different groups in relation to the *P* value (<0.05). Iron elements were found in the predentin and circumpulpal dentin areas, and the results obtained showed a statistically significant difference between dentin tissue from patients with diabetes and those without. Individuals with Type 2 DM are prone to present iron precipitates in predentin and circumpulpal dentin tissue. Few iron elements were found in dental organs of individuals without DM.

## 1 INTRODUCTION

Diabetes mellitus (DM) is a frequent worldwide disease. There are currently more than 46 million people who suffer this disease in North America and the Caribbean, and these figures are increasing (International Diabetes Federation, 2017). Type 2 DM is a metabolic syndrome that generally develops in adults. It is characterized by a hyperglycemic state that depends or not on the administration of insulin and that causes diverse consequences in the individuals who suffer the disease (American Diabetes Association, 2014). Iron is a fundamental mineral that promotes adequate cell maintenance and proliferation, and it also participates in oxygenation processes in all cells of the body (Oexle, Gnaiger, & Weiss, 1999). There are multiple benefits when there are optimal levels of iron, but when the human body has an elevated amount of iron, this is associated with a greater risk of glucose metabolism disorders (Lee, Kim, & Kim, 2011) and Type 2 DM (Bao, Rong, Rong, & Liu, 2012). One of the effects of iron is to generate reactive oxygen species and nitrogen which can produce oxidative stress that releases free radicals that induce cell damage especially in pancreatic beta cells which are susceptible to oxidative stress (Asmat, Abad, & Ismail, 2016).

The human hemochromatosis protein controls the interaction between the transferrin receptor and transferrin regulating the absorption of iron by the intestine. When it binds to the transferrin receptor, it reduces its affinity for transferrin, and when it reaches the pancreas, there is deposition of iron in the islets. This causes the production of reactive oxygen species that produce β‐cell failure and insulin resistance with this being a possible cause of DM (Simcox & McClain, 2013).

Dentin is a mineralized avascular tissue that forms the bulk of the tooth. It is live tissue that produces the shape of the tooth. Dentin is collagen that is mineralized after organization of the collagen matrix in predentin. Predentin is unmineralized dentin that is 20‐ to 30‐μm wide and that is found adjacent to the pulp surface. It represents the formation of dentin before its calcification and maturation (Tjäderhane & Haapasalo, 2009).

There is an association between iron overload and Type 2 DM. It has been found that moderate increases in iron are associated with glucose and insulin elevations (Bao et al., 2012). The nonmineral structure of bone (osteoid) and dentin are similar; therefore, there is a relationship with iron deposits in dentin, specifically in predentin (Hess & Villanueva, 1982). Potassium ferrocyanide is a histological stain that is commonly used to detect the presence of iron in tissue samples (Crudo, Erramouspe, Sueldo, & Arias, 2016).

The objective of this research is to determine if there is an association between DM and the presence of iron precipitates (Fe^2+^) in dental structure.

## 2 METHODS

For this study, third molars from each subject were analyzed. The teeth were extracted for diverse reasons in individuals over 18 years and less than 40 years of age with and without Type 2 diabetes. Teeth had to be healthy with Grade 1 caries with enamel restoration and coronary integrity. Exclusion criteria were teeth with trauma, with extensive restoration, without coronary integrity, and with external or internal radicular resorption. Elimination criteria included teeth with fractures during the study or any failure during the experimental process. This study was previously approved by the Ethics Committee with registration number SPSI 0106113—folio 00113.

A total of 40 upper and lower uni and multiradicular total or partially erupted third molars were extracted and later placed in a Falcon tube with artificial saliva and a drop of hypochlorite at 37°C. The teeth were divided into two groups of 20 third molars each: experimental (A) and control (B). They were later embedded in crystal resin, placing each specimen in a heavy silicon mold to form cubes and later perform hard tissue slices.

Tooth slices were made from the upper portion of the clinical crown in a coronal manner to the apical portion separating both parts and obtaining 1‐mm thick flat slices; this was done with a water‐cooled diamond disc with a Marathon III electric micromotor (Saeyang Microtech Co., Shanghai, China) to obtain a highly polished surface.

A solution was prepared by mixing equal parts of 20% hydrochloric acid (Sigma‐Aldrich, Inc. St. Louis, MI, USA) and 10% potassium ferrocyanide (K_4_Fe(CN)_6_·3H_2_O; Sigma‐Aldrich, Inc.) diluted in distilled water for 40 min obtaining half a liter of solution in a glass receptacle. Each slice was placed individually in a screw‐top glass container. Afterwards, the slices were washed in distilled water three times each for 5 min removing the excess liquid of the slices. After staining, the slices were placed on glass slides for observation under an optic microscope (LSM510, Carl Zeiss Co. Ltd., Jena, Germany). Images of the specimens were observed at a magnification of 4x/0.10/0.17, 10/0.25/0.17, and 40x/0.65/0.17, identifying the areas of tissue and visually counting the number of iron precipitates and obtaining photographs in each magnification with a Canon EOS Rebel T5 camera (Canon U.S.A., Inc. Huntington, NY, USA).

### 2.1 Statistical analysis

The data obtained were captured in an Excel 2016 database with which frequency tables of the variables capillary glucose/iron tissue dental precipitates were compared with the remaining variables. A nonparametric Spearman correlation test was applied to the results to find an association between the original variables. The Mann–Whitney U test was applied to find differences in the means of nonparametric variables in the two groups with a *P* < 0.05 being significant.

## 3 RESULTS

The results of the experimental and control group in the clinical crown are shown in Table 1. No significant difference was found between the experimental group and the control with regard to age (*P* = 0.295); however, a significant difference was found in capillary glucose (*P* < 0.001) and in the predentin and circumpulpal dentin tissue (*P* < 0.001), individually and as a group.

**Table 1:** Analysis of the experimental and control group with regard to iron precipitates found and observed in dental tissue of the clinical crown

**Group**	**Specimen (crown)** a		**Mann–Whitney U**	***P* value**
	**Experimental**	**Control**		
Age	22.43	18.43	161.5	0.295
Capillary glucose (mg/dL)	30.40	10.60	2.0	<0.001
Dentin layer	23.00	18.00	150.0	0.019
Predentin	27.13	13.88	67.5	<0.001
Circumpulpal dentin	26.18	14.55	81.0	<0.001
Dental tissue (mean of 3 areas)	25.18	14.83	86.5	0.001

a Data are presented as means.

The results of the variables of the experimental and control group in dental tissue of the root are shown in Table 2. There was no significant difference between the experimental group and the control with regard to age (*P* = 0.295). A significant difference was found in capillary glucose (*P* < 0.001) and the respective tissue areas of the dental root both individually and as a group (*P* < 0.001).

**Table 2:** Analysis of the experimental and control group with regard to iron precipitates found and observed in dental tissue of the root

**Group**	**Specimen (Root)** a		**Mann–Whitney U**	***p* value**
	**Experimental**	**Control**		
Age	22.43	18.58	161.5	0.295
Capillary glucose (mg/dL)	30.40	10.60	2.0	<0.001
Dentin layer	24.00	17.00	130.0	0.004
Predentina	27.50	13.50	60.0	<0.001
Circumpulpal dentin	27.03	13.98	69.5	<0.001
Dental tissue (mean of three areas)	27.30	13.70	64.0	<0.001

a Data are presented as means.

**Figure 1:**
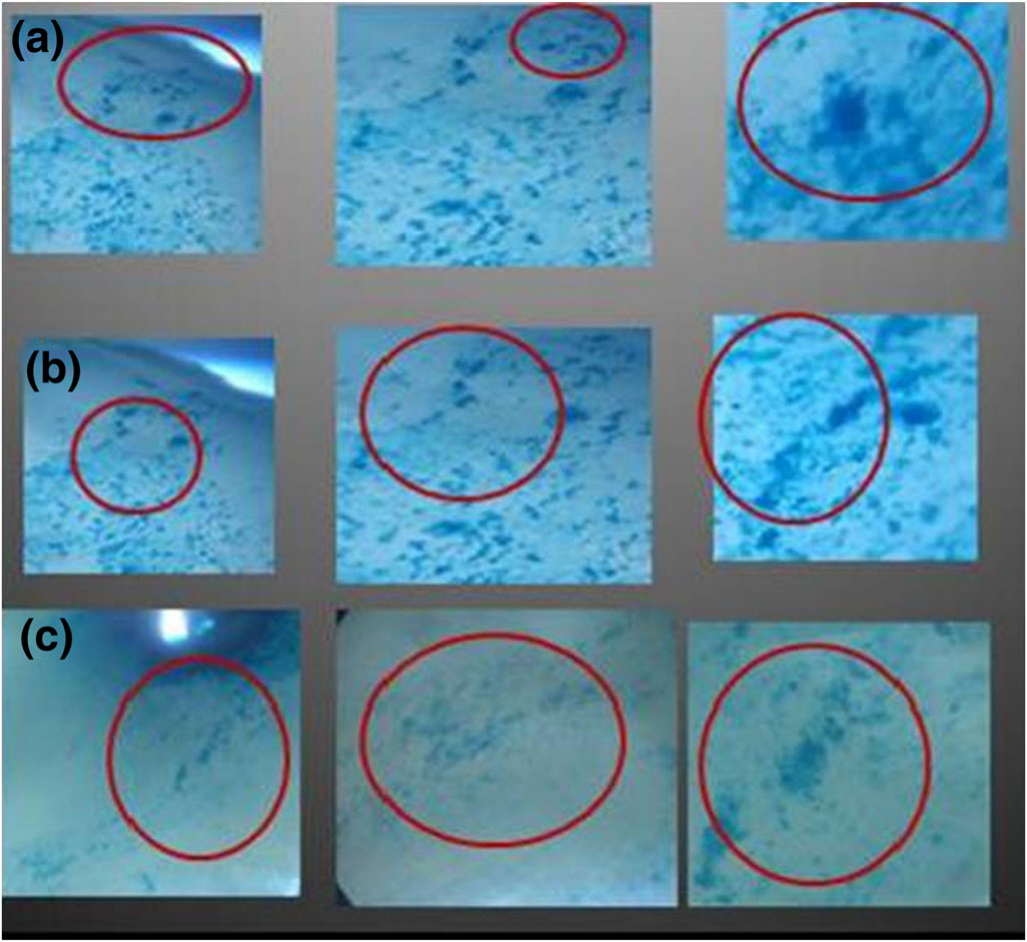
Photomicrograph from experimental group, left 4x; middle, 12x; right 40x; circled areas show a greater concentration of precipitates. (a) Mantle dentin, crown, and amelodentinal junction. (b) Predentin, crown, and amelodentinal junction. (c) Crown circumpulpal dentin

**Figure 2:**
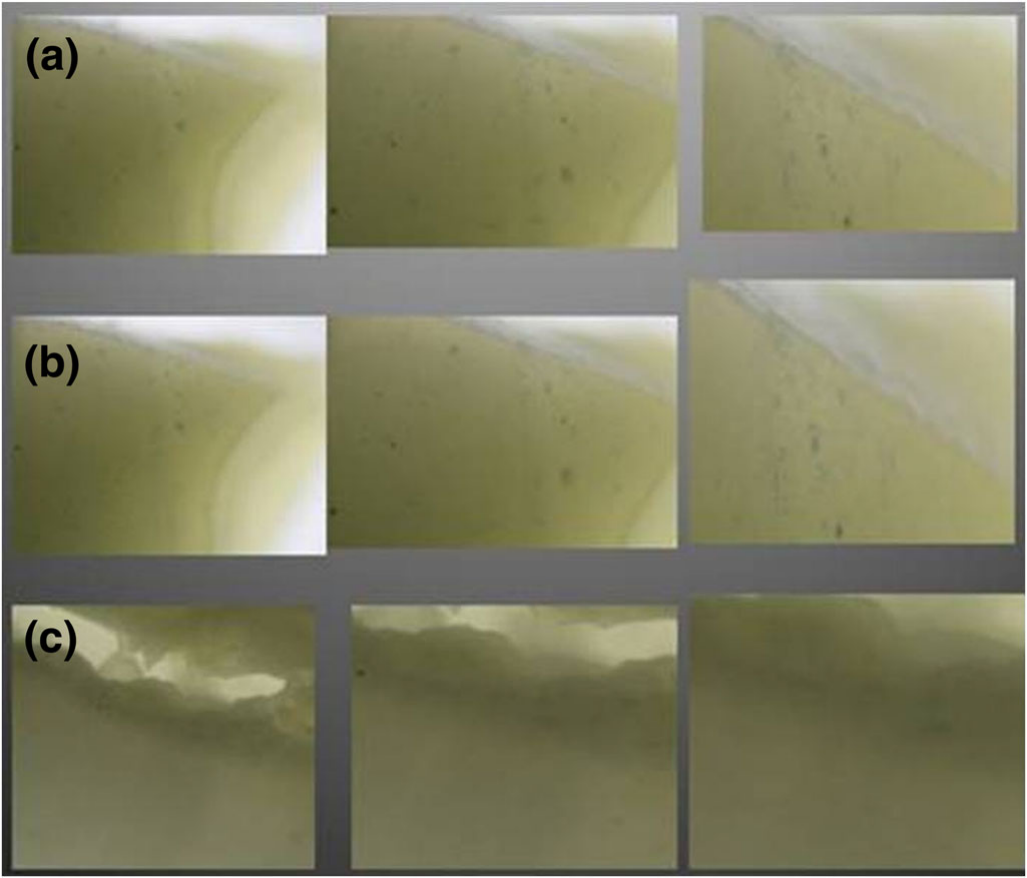
Photomicrograph from control group, left 4x; middle, 12x; right 40x. (a) Mantle dentin, crown and amelodentinal junction. (b) Predentin, crown and amelodentinal junction. (c) Mesial root circumpulpal dentin

With regard to the presence of iron precipitates in the areas of dental tissue both in the clinical crown and in the dental root, a significant amount was found in the areas of the predentin and circumpulpal dentin, with this being less in the dentin layer (Figure 1a–c) in contrast to the same areas in the control group where no precipitates were seen (Figure 2a–c).

The iron necessary for the formation of dentin as well as for the diverse systemic effects caused by DM is deposited in dental tissue with this being greater in the predentin and circumpulpal dentin areas. In relation to negative associations in the group variables, a figure of −85% was obtained in the experimental group versus the control group in high capillary glucose levels.

## 4 DISCUSSION

It was possible to identify iron precipitates in dental tissue in individuals with DM using 10% potassium hexacyanideferrate. Iron elements were found in a greater concentration in circumpulpal dentin and in predentin with a statistically significant difference between the area of the dental root and the area of the clinical crown in comparison with a control group of people without diabetes. This demonstrates that the observation of iron precipitates in hard tissue is associated with patients with Type 2 DM with high glucose levels.

Elevated iron stores have been associated with a risk of Type 2 diabetes. Higher serum ferritin levels were more prevalent in a South Korean population with metabolic syndrome and DM in the 2008 Korean National Health and Nutrition Examination Survey (Lee et al., 2011) demonstrating a positive association between elevated iron stores (measured by serum ferritin levels) and the prevalence of metabolic syndrome and DM after adjustment for age, sex, educational level, smoking, alcohol intake, and body mass index. This has also been seen in patients with hereditary hemochromatosis and transfusional iron overload as in beta thalassemia major and bone marrow transplantation (Simcox & McClain, 2013). The pathogenesis of diabetes has been associated with elevated iron deposits (Thomas, MacIsaac, Tsalamandris, & Jerums, 2004).

Fernandez‐Cao et al. (2013) carried out an observational cohort analysis of individuals without diabetes but with cardiovascular risk followed for 1–8 years in the PREDIMED Trial. Of the initial sample, 12.2% developed diabetes with a median follow‐up of 4.8 ± 1.3 years. These participants had a greater intake of total iron and heme iron in the form of meat consumption. Red meat, especially processed meat, has been implicated, in addition to Type 2 diabetes, in cancer, Alzheimer's disease, and cardiovascular disease.

Elevated iron stores cause insulin resistance and possibly defective secretion of insulin. Patients with DM and elevated serum iron concentrations have higher glucose levels (Misra, Bhatter, Kumar, Gupta, & Khan, 2016).

Another problem with iron deposits in dentin is that they can act as an intrinsic stain altering the color of teeth (Bonilla, Mantín, Jiménez, & Llamas, 2007). The clinical complications of intrinsic stains of dentin can compromise aesthetics and dentin adhesion capabilities. Because intrinsic stains mainly affect aesthetics, the intensity of the pigment must be identified. This alteration ranges from mild to severe. Diagnosing the magnitude of the damage as well as the etiological factor will determine the appropriate treatment to achieve predictable results (Haro‐Velastegui, 2012).

Because of the scarce information regarding diabetes in relation to the hard tissues of dental organs, more research is needed in the subject because these changes are not observed by simple clinical inspection and often not even radiographically; however, the reports in this study show that there are adverse processes that can only be observed by microscope in a hard tissue specimen of an extracted dental organ.

More research on this subject could be useful to prevent early stage Type 2 DM because these precipitates are present in people with diabetes and in people susceptible to diabetes. This finding could be useful to identify susceptibility to DM and to confirm the disease (Hess & Villanueva, 1982).

Based on the results in this study, the presence of iron precipitates in dental tissue in the predentin and circumpulpal dentin layers of the clinical crown, and the tooth root was statistically significant in study subjects with Type 2 DM in which hard dental tissue slices were stained to identify iron precipitates. It would be interesting to see if this also correlates with serum ferritin levels; however, further research is needed to clarify this point.

## 5 CONCLUSIONS

DM affects some of the independent variables of the crown. Staining with 10% potassium ferrocyanide identified iron precipitates in dental tissue in individuals with Type 2 diabetes. Iron precipitates were found in dental tissue with this being greater in individuals with high blood glucose levels.

## DATA AVAILABILITY

Data are available by contacting Dr. Miguel Angel Ortiz‐Arrambide, email: odmaoa@hotmail.com.

## COMPETING FINANCIAL INTERESTS

No specific funding was received for this study.

## CONFLICT OF INTEREST

The authors declare that they have no conflicts of interest in this study.

## AUTHOR CONTRIBUTIONS

Miguel Angel Ortiz‐Arrambide—Project conception and design, acquisition, analysis, and data interpretation, drafted the manuscript. Karla Isabel Juarez‐Ibarra—Reviewed data acquisition and analysis, the intellectual content, and the draft of the manuscript and approved the final version of the manuscript. Guadalupe Ismael Malagón‐Santiago—Reviewed data acquisition and analysis and provided statistical support. Norma Cruz‐Fierro—Reviewed data acquisition and analysis and approved the final version of the manuscript. Myriam Angelica De La Garza‐Ramos—Reviewed data acquisition and analysis, the intellectual content, helped draft the manuscript, and approved the final version of the manuscript.
